# Flexural Strength Prediction Models for Soil–Cement from Unconfined Compressive Strength at Seven Days

**DOI:** 10.3390/ma12030387

**Published:** 2019-01-26

**Authors:** Alaitz Linares-Unamunzaga, Heriberto Pérez-Acebo, Marta Rojo, Hernán Gonzalo-Orden

**Affiliations:** 1 Department of Civil Engineering, University of Burgos, 09001 Burgos, Spain; mrarce@ubu.es (M.R.); hgonzalo@ubu.es (H.G.-O.); 2 Mechanical Engineering Department, University of the Basque Country UPV/EHU, 48013 Bilbao, Spain; heriberto.perez@ehu.eus

**Keywords:** soil–cement, cement treated materials, cement treated base materials, flexural strength, unconfined compressive strength, long term, short term

## Abstract

Soil–cement is an environmentally friendly road construction technique for base and subbase materials, which allows employing soils placed in the right-of-way of the road or in the surroundings, by improving its engineering properties. With this technique, it is possible to reduce the over-exploitation of quarries, the necessity of landfills and the pollutant gas emission due to the reduction of aggregate fabrication and transport. The manufacturing of soil–cement is generally controlled by means of the Uniaxial Compressive Strength (UCS) test at seven days, according to the regulations of each country. Nonetheless, one of the properties that best defines the performance of soil–cement is the Flexural Strength (FS) at long term, usually at 90 days. The aim of this paper is to develop new equations to correlate the UCS and the FS at long term and the UCS at seven days and at 90 days. Obtained results validate the proposed models and, hence, the flexural strength can be predicted from the Uniaxial Compressive Strength at seven days, allowing, if necessary, correcting measures (recalculation or rejection) in early stages of the curing time to be taken.

## 1. Introduction

At present, there is an international consensus about the necessity for a sustainable development, which makes researchers look for more environmentally friendly solutions, focused on both managing the natural resources more efficiently and reducing the carbon footprint [1,2,3,4,5,6,7].

Road pavements consist of various materials layers, which are generally referred to as surface, base course, and subbase, built over a compacted subgrade. There are two main types of pavement surfaces: Portland cement concrete and hot-mix asphalt, also called asphalt concrete [8]. Below the surface, the base and subbase layers provide structural support for the pavement system [9]. These layers may include either aggregate or treated base and subbase layers. The subgrade is generally a local aggregate material, but sometimes the top of the subgrade is stabilized with cement or lime.

In bases and subbases, various soils or granular materials are deployed, but they may have insufficient properties [10,11,12], such as low bearing capacity, which can cause pavement distresses and reduce pavement life [13,14,15,16]. To improve the properties of base or subbase, some stabilization agents are usually added to the soils. The most widely employed additives are cement, bitumen and lime. Among these improved base and subbase materials, cement-treated materials (CTM) achieve quite a high stiffness and strength, providing a good performance to the pavement structure, maintaining for longer its serviceability and riding comfort. Moreover, cement-treated materials are said to distribute loads over a wider area and, hence, reduce the stress on the subgrade [6,16,17,18].

One of the materials included in the cement-treated materials is soil–cement [14,15,16,19,20,21,22]. This technique can be produced in a manufacturing plant, where employed aggregates can totally or partially come from a recycling method [1,2,3,23,24,25] or in situ, using devices similar to in the subgrade stabilization procedure [19,20,26], taking advantage of the engineering properties of the soil that is placed in or near the road.

In both cases, it is an environmentally friendly technique. Treating materials with cement allows a reduction in the over-exploitation of quarries, the use of landfills and greenhouse gas emission due to the lower necessity of aggregate crushing and transport.

With regard to the characteristics of soil–cement, as any layer treated with cement, they depend on multiple factors, such as the amount of added cement, the quantity and type of fine aggregate of the soil, the moisture content, the curing process and the age of the compacted material [3,6,15,16,17,27,28,29].

The Unconfined Compressive Strength (UCS) test at short term is the most used test to verify that the soil–cement was manufactured correctly [30,31,32,33,34,35]. On the other hand, a better characterization of the long-term performance is obtained by means of the Flexural Strength (FS) tests, and, more specifically, the four-point flexural beam test [17,18,36,37,38,39].

To conduct the FS tests, prismatic samples are needed. However, manufacturing a prismatic specimen with an acceptable quality is not an easy task because it depends on the qualification and experience of the testing team [40,41]. Consequently, long-term values are usually estimated from indirect tests, such as the Unconfined Compressive Strength (UCS) and the Indirect Tensile Strength (ITS), which are more standardized [16,17,20,42,43,44].

Recent research shows that, apart from the values obtained in the indirect tests, other variables influence the FS values, e.g. the cement content, the density and the moisture content of the mixture [17].

The aim of this paper is to establish a relationship between the FS and the UCS at long term by means of multiple lineal regression models, verifying the adequacy of the results to existing formulae and, if necessary, proposing a new relationship that better fits the values. Simultaneously, a relationship between the FS at long term and the UCS in short term is suggested. Therefore, after carrying out the UCS test at seven days, which is employed for accepting, recalculating the entire pavement structure or rejecting the manufactured soil–cement, it is possible to know its performance at long term, i.e., simulating its performance under cyclic tensile loads, which are the main reason for failure in materials treated with cement [16,17,20,45,46].

The article is organized as follows. Section 2 defines the employed materials in soil–cement and mix design. Section 3 shows the experimental procedure. Obtained results are presented and discussed in Section 4. Finally, a summary and the conclusions are given in Section 5.

## 2. Materials and Mix Design

### 2.1. Materials

**Figure 1:**
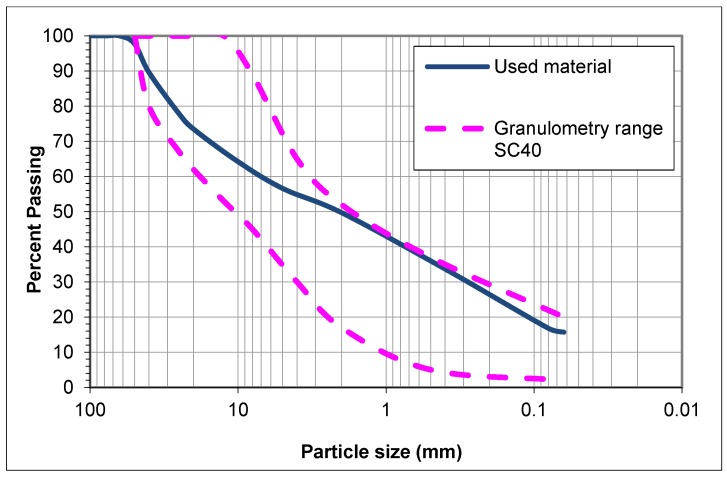
Soil granulometry and granulometry range for SC40.

The employed material was a soil from the north of Spain with a maximum aggregate size of 40 mm. Figure 1 shows the granulometry of the material, which is inside the range of the SC40 (soil–cement with a maximum aggregate size of 40 mm). According to the Spanish standards [32], this is used for soil–cement as a subbase material for any type of road.

Regarding the characteristics of the soil, it was not plastic [47], did not have organic material [48] of soluble sufate [49], the sand equivalent test gave a value of 20 [50] and it was classified as SP-SM following the Unified Soil Classification System (USCS) [51].

The cement used was CEM IV B/V 32.5 N [52]. It is a widely used cement type in roads for soil stabilization and soil–cement because it has a lower thermal shrinkage and maintains the workability of the soil–cement for a longer period due to the low quantity of clinker, high quantity of additives and moderate strength, mainly at short term [20]. Table 1 shows the characteristics of this cement. 

### 2.2. Mix Design

The main parameters to characterize soil–cement are the maximum dry density and the optimum moisture content (both obtained by means of the Modified Proctor test), the amount of cement and the minimum compressive strength at seven days. As the densities obtained vary as a function of the soil type (and other variables), instead of establishing a minimum density for cement-treated materials, highway administrations require a percentage of the maximum dry density obtained with the Modified Proctor test. The determination of maximum dry density and optimum moisture content was conducted with cylindrical samples following the UNE 103-501-94 [53], whose prescriptions are very similar to the ASTM D1557-12 [54].

The amount of cement added to the mixture is what guarantees the minimum compressive strength at seven days, as indicated in Table 2.

**Figure 2:**
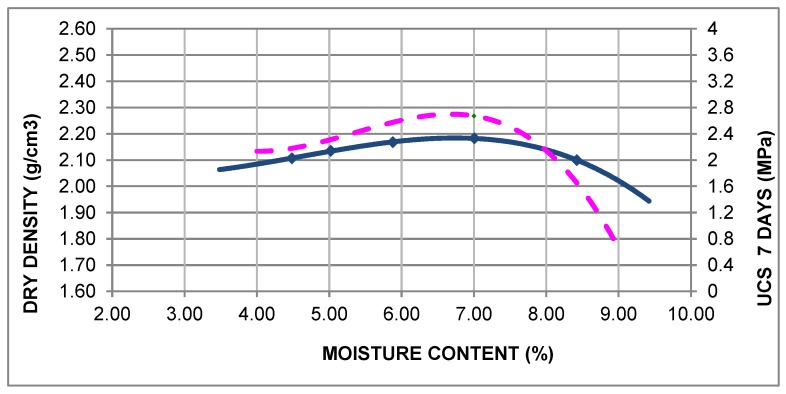
Modified Proctor density of the soil–cement with a 3.5% content of cement.

For this analysis, a mix containing 3.5% cement was employed, which had a UCS at seven days of 2.67 MPa, a maximum dry density of 2.18 g/cm^3^, and an optimum moisture content of 7.0%, as it can be seen in Figure 2.

## 3. Experimental Procedure

As previously stated, the manuscript aims to analyze the flexural strength performance of a soil–cement mix for long-term characterization (90 days), according to the results from some tests. The paper also aims to suggest a new relationship between the FS and the UCS at short term (seven days) since this value is used as a reference to accept soil–cement in fieldwork.

**Figure 3:**
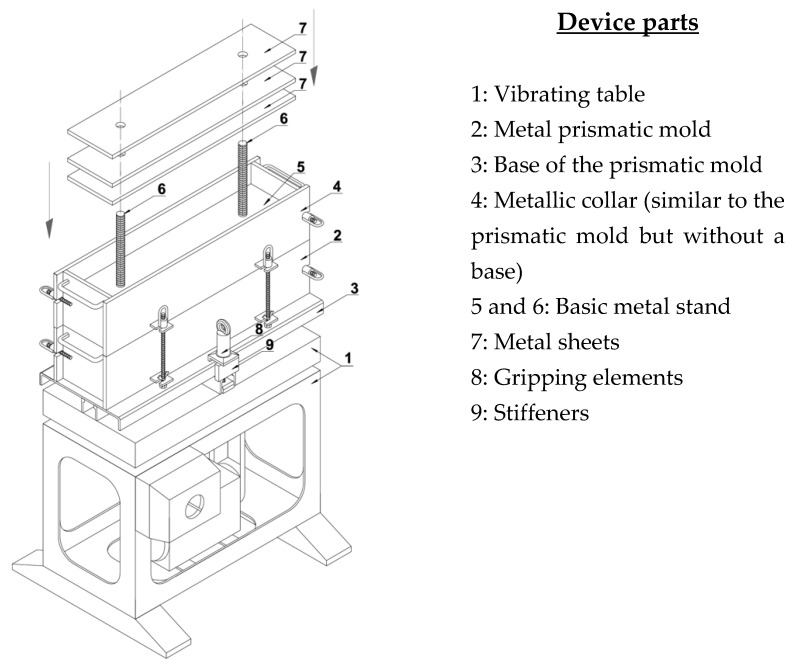
Device for compacting prismatic specimens.

With this aim, prismatic samples were manufactured, which are required for the FS test, following the procedure described by Linares-Unamunzaga et al. [55], with its specific device (Figure 3). This procedure can be repeated with the devices that are usually available in any soil laboratory. The mixture, with 3.5% cement and a 7% moisture content, was placed in three layers in the mold, with inside dimensions of 15 cm × 15 cm × 60 cm (Element 2 of Figure 3). Each layer was compacted by placing five metal sheets (Element 7 of Figure 3) over the metal stand (Elements 5 and 6 of Figure 3) and vibrating it by means of a vibrating table of 40 Hz (Element 1 of Figure 3) for 20 s.

Following this procedure, it was possible to achieve compaction densities not less than 98% of the maximum dry density obtained with the Modified Proctor as the Spanish regulations established [32].

Once the specimens were manufactured, they were placed in the curing room at 20 ± 2 °C and ≥95% RH (relative humidity) [56]. After 24 h, the metal prismatic mold (Element 2 of Figure 3) was removed. However, the base of the mold (Element 3 of Figure 3) was removed after seven days to guarantee a minimum resistance and to avoid breaking the sample as a consequence of operation.

At a curing age of 90 days, the four-point flexural beam test was conducted, since it is considered the best test to replicate the long-term performance of the cement-treated layers under traffic loads [18,36,39,57,58].

This test was conducted following the standard UNE-EN-12390-5 [59]. The rollers below the specimen were placed at a distance of 45 cm (three times the height of the specimen), and the rollers over the specimen at a distance of 15 cm (the height of the specimen). The applied load was transmitted by means of a plate between the specimen and the rollers over it. The standard allows a constant increasing tension in the range of 0.04–0.06 MPa/s. An increasing tension of 0.04 MPa was selected to avoid collapsing the sample, since the load was applied in the slowest way. The end of the test was programmed when a reduction of the strength of 5% was detected.

After the FS test, the specimen was divided in two prismatic parts. The UCS test was conducted over each of these parts, following the standard UNE-EN 13286-41 [60], with a load speed in the range interval of 0.1 ± 0.1 MPa/s [61]. In this case, to simulate the behavior of a cubic sample, an auxiliary metal sheet of 15 cm × 15 cm was placed between the lower side of the prismatic sample and the lower plate and another one with similar dimensions between the top side of the sample and the top plate. Hence, it was possible to obtain a uniform tensile distribution in a cube of 15 cm × 15 cm × 15 cm.

With the results from both tests, mathematical models were developed to correlate both long-term strengths (FS and UCS) and they were compared with the ones proposed by other authors [17,20,42,44].

Firstly, a simple linear regression model was tested.

- Model 1. UCS is the independent variable and the FS is the dependent variable. Model 1 has two versions: an “a” version, without an intercept, and a “b” version with an intercept.

Another important value that is found in the regulations is the density, which was also considered in the models, giving an answer to the demands of the scientific community [16]. Particularly, in the Spanish case, the density must not be below 98% of the maximum density obtained with the Modified Proctor procedure [32]. Considering the low variability range of the required density in the road agencies, i.e., from 95% to 100% of the maximum dry density obtained in the Modified Proctor procedure [30,31,32,33,34], different multiple regression models were tried. In all of them, the “a” version does not consider the intercept but the “b” version does.

- Model 2. It includes a new independent variable that represents the percentage of the difference between the compaction density of the sample and the maximum dry density value.
- Model 3. It includes a dummy variable as an independent variable, which has a value of 1 if the obtained density is greater than or equal to the maximum dry density of the Modified Proctor and has a value of 0 if the density is less than the maximum dry density.
- Model 4. It includes two dummy variables as independent variables. The first one has a value of 0 if the obtained density is less than or equal to the maximum dry density minus 1% and 1 if the density is over this value. The second dummy variable has a value of 0 if the density is less than or equal to the maximum density plus 1% and 1 if the density is over that value.
- Model 5. It includes two dummy variables as independent variables. The first one has a value of 0 if the obtained density is less than or equal to the maximum dry density minus 1% and 1 if the density is greater than this value. The second dummy variable has a value of 0 if the density is less than or equal to the maximum density and 1 if the density is greater than that value.

Finally, three different models were tested by means of the Cobb–Douglas production function. This function allows obtaining simple models that can verify the lineal condition, inherent to the lineal regression models, but it requires a previous transformation using logarithms.

- Model 6. It only uses the FS and UCS variables
- Model 7. It includes a new independent variable, which is the difference between the obtained density and the maximum density from the Modified Proctor test, in percentage.
- Model 8. It includes a dummy independent variable, with a value of 1 when the compaction density is greater than or equal to 100% of the Proctor Modified density and 0 when it is less than the Proctor Modified density.

when linear regression models are used, it is necessary to consider some assumptions [62]:

- Linearity of the relationship between the dependent variable and the independent variables. This can be checked by the analysis of variance (ANOVA) analysis.
- Homoscedasticity. This implies that the variance of error term is constant across all values of the independent variables. This is verified by means of a plot of the standardized residuals obtained against the predicted standardized residuals and observing that there is no pattern on it.
- Normality. This means that the error is normally distributed, which can be verified by the Kolmogorov–Smirnov test.
- Each observation is drawn independently from the population, implying that errors are independent from each other. This is checked with the Durbin–Watson test.

In the case of multivariate models, the selection of the best model was carried out from a ranking process with the following criteria:

- Verifying the signs of the variables.
- Testing the significance of the variables by means of the t-Student test.
- Analyzing the dummy variables, guaranteeing the non-linearity if various ones are introduced at the same time.
- Other criteria, such as the variables employed by each model and the regression coefficient R^2^.

Finally, for validating the soil–cement, it is compulsory to achieve a minimum UCS value at seven days. This value is usually obtained from testing cylindrical specimens and, therefore, it can be useful to establish the increasing evolution of the UCS value over time. This way, it would be possible to estimate the flexural strength at long term from the compressive strength test at short time. Hence, when manufacturing soil–cement, it would be possible to establish corrective measures if the obtained FS is not the expected one. With this aim, ten batches were produced and from each batch the following samples were manufactured:

- A prismatic sample with dimension 15 cm × 15 cm × 60 cm, following the same procedure explained for the previous prismatic samples. The only difference was that a flexible plastic film was placed perpendicular to the length of the mold to manufacture two separated prismatic samples to be tested at 7 and 90 days, respectively.
- Three cylindrical samples with dimension ∅15 × 18 compacted in three layers were tested at 7, 28 and 90 days, respectively.

This way, it was possible to correlate the UCS of the cylindrical specimens with the UCS of the prismatic samples at 90 days since the first ones were used to validate the manufacturing of the soil–cement at seven days and the second ones were employed for the calculation of the FS by the four-point flexural beam test.

## 4. Results and Discussion

### 4.1. Flexural Strength Test

**Figure 4:**
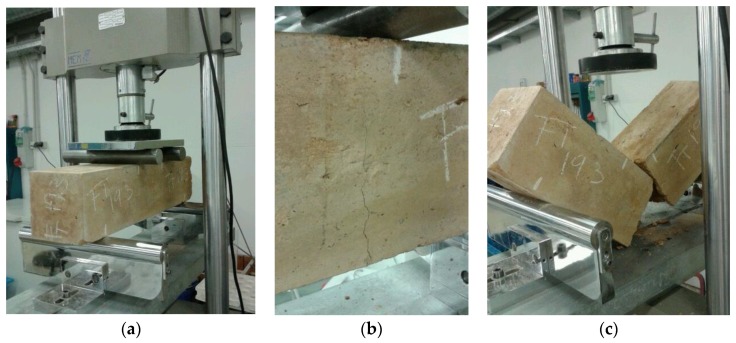
FS test: (**a**) placing of the sample; (**b**) breaking of the sample; and (**c**) separation of the prismatic parts.

The flexural strength (FS) was determined by the four-point flexural beam test [59,63] (Figure 4a). By means of this test, the samples were broken in a central section of 15 cm × 15 cm × 15 cm, where the bending moment was considered to be constant. Hence, the failure was verified to be produced by exceeding the tensile strength of the weakest section in the lower side of the specimen (Figure 4b).

In total, 63 FS tests were performed. It must be pointed out that the dimensions of the used samples, the weight of the mold and the compaction density made the operations difficult with the samples, since each one weighted around 54 kg. This value was reduced to 30 kg when the test was carried out due to the removal of the mold and the moisture lost during the curing time. Nevertheless, these dimensions of the specimens were necessary for the selected maximum aggregate size (40 mm) to fulfill the requirements of the Spanish regulations [32] for manufacturing soil–cement.

According to the statistical analysis of the results of the four-point flexural beam test, the specimens had an average flexural strength of 0.86 MPa, with a standard deviation of 0.10 MPa. The median was 0.87 MPa with an interquartile range of 0.12 MPa. The difference between the maximum and the minimum value was 0.385 MPa. The skewness and kurtosis coefficients showed a negative skew (to the left) and platykurtic distribution [64,65].

### 4.2. Unconfined Compressive Strength Test

**Figure 5:**
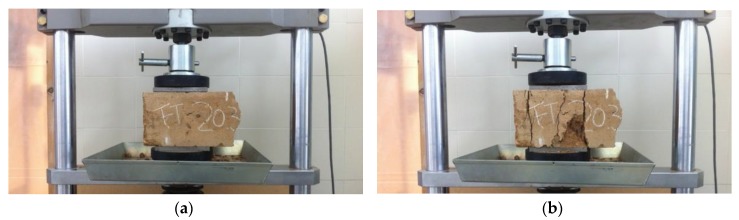
UCS test: (**a**) placing of the prismatic part; and (**b**) breaking of the specimen.

After the FS test, the specimen was divided into two prismatic parts whose breaking sides were defined by the generated cracking. The UCS test was conducted over each of these parts, following the standard UNE-EN 13286-41 [60]. To simulate the behavior of a cubic sample, two auxiliary metal sheets of 15 cm × 15 cm were placed between the lower side of the prismatic sample and the lower plate and between the top side of the sample and the top plate. Hence, it was possible to obtain a uniform tensile distribution in a cube of 15 cm × 15 cm × 15 cm, guaranteeing that the breaking occurred in that cube (Figure 5b). The test was programmed to finish when a 5% strength reduction was detected, avoiding the total failure of the sample.

According to the statistical analysis of the results from the UCS test, the specimens had an average UCS of 4.64 MPa, with a standard deviation of 0.69 MPa. The median was 4.70 MPa and the interquartile range was 0.87 MPa. The difference between the maximum and the minimum value was 3.425 MPa. The skewness and kurtosis coefficients show a negative skew (to the left) and a mesokurtic distribution [64,65].

### 4.3. Flexural–Compressive Strength Relationship Models

The advantage of conducting the UCS tests over the parts resulting from the FS tests was that, despite the heterogeneity of the material, the results could be compared, making it possible to correlate obtained results. A total of 125 observations were employed in this study (one part of a prismatic sample was accidentally broken).

The eight proposed models in Section 3 were tested with the obtained values. Only the models that provided the best results are commented on below.

The analysis of the simple linear regression for the values of UCS and FS at long term (Models 1a and 1b) is shown in Table 3.

According to the results in Table 3, and taking into account the *p*-values for the slope in both cases, it can be stated that there is a positive linear correlation between the variables UCS and FS. The *p*-value of the Kolmogorov–Smirnov test indicates that the residuals have a normal distribution with a significance level of 95% in both cases. The plot of the residuals vs. the predicted residuals confirms the homoscedasticity of the models. The independence of the observations is not assured by the Durbin–Watson statistic, but the plot of the residuals does not show any pattern. Therefore, both models could be used to represent the relationship between the UCS and the FS.

However, Model 1b has a better R^2^ and a lower estimated standard error. Moreover, the intercept is significant (*p*-value < 0.0001) [64,65].

**Figure 6:**
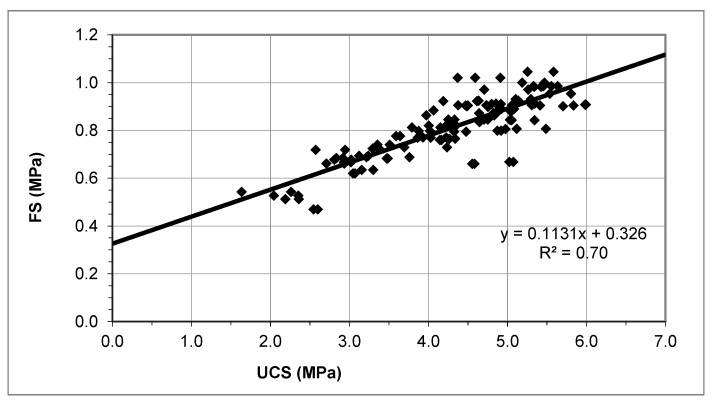
Relationship between UCS and FS at long term.

Consequently, the relationship between the UCS and FS in the long term can be modeled from Model 1b by means of Equation (1) (Figure 6).

(1) FS=0.1131×UCS+0.3261

Considering the density an independent variable in the relationship, from the originally proposed models, the ones that give a better result are Models 3a and 3b (Table 4).

As observed in Table 4, both models confirm the linearity of the proposed model with a significance level over 90%. The dummy variable in both models has a value of 1 if the density is over or equal to the maximum dry density obtained in the Modified Proctor test and 0 when it is lower than this value.

Both models show a similar fitted R^2^. However, the mean standard error is lower in Model 3b and, as the intercept is significant, it should not be omitted [64,65]. Additionally, the dummy variable is more significant in Model 3b.

Therefore, the relationship between UCS and FS, considering the compaction density percentage, could be expressed, using the Model 3b, by Equation (2):

(2) FS=0.1124×UCS+0.0319×Dummy +0.3230

When the relationship is determined following the production function of Cobb–Douglas, after the statistical analysis, the models with better results are Models 6b and 8b, which were obtained by applying natural logarithms. Their results from the statistical analysis are presented in Table 5.

As observed in Table 5, apart from the coefficient value for the dummy variable, the other coefficients have similar values and they are all significant (*p*-value < 0,0001). However, the *p*-value of the dummy variable in Model 6b is around 0.10, which means that it only has a 90% significance. Therefore, Equation (3) can be employed to model the relationship between the UCS and the FS considering the compaction density obtained by means of the product function of Cobb–Douglas from Model 6b.

(3) $$\mathrm{FS}=e^{-1.05}\times {\mathrm{UCS}}^{0.58}$$

From the three selected models presented in Equations (1)–(3), and according to the number of used variables and the value of R^2^ (fitted R^2^ in the case of the multiple regression), it is recommended to estimate the FS at long term after the following priority order:

(2) FS=0.3230+0.1124×UCS+0.0319×Dummy

(3) $$\mathrm{FS}=e^{-1.05}\times {\mathrm{UCS}}^{0.58}$$

(1) FS=0.3261+0.1131×UCS

The problem with Equations (1)–(3) is that they do not fulfill the basic assumption that, if a sample has no UCS, it will have no FS as well. Hence, it is necessary to establish the minimum UCS value from which Equations (1)–(3) are valid. To gather all the possible cases of UCS, this value should not be below the minimum UCS at seven days required by the different regulations [30,31,32,33,34]. Therefore, Equations (1)–(3) can be applied if the UCS is over 1.5 MPa.

### 4.4. Comparison with Other Proposed Models

There are some relationships to correlate UCS and FS at long term in the literature (Table 6).

Among them, the formulae proposed by Kersten [44], IECA-CEDEX [20] and Lim and Zollinger [42] have the form of Equation (10).

(10) FS=a×UCS

where “a” is an experimental parameter that has a value of 0.2 [44] or between 0.2 and 0.25 [20,42]. For the average value of UCS of this study, 4.64 MPa, and applying Equation (10) and depending on the value of the “a” coefficient, the FS values will be over the average value (0.86 MPa vs. a range between 0.93 and 1.16 MPa). Employed soil–cement gives a value of 0.16 for the “a” coefficient, below the minimum value indicated by these authors.

Ismail et al. [17] proposed some recent equations, which suggest various multivariate relationships. From them, Equations (6) and (7) can be compared with the data from this study, where UCS is the unconfined compressive strength (MPa), FS is the flexural strength (MPa) and t is the curing time (days). If UCS is calculated by Equations (6) and (7), supposing an average value of 0.86 MPa for FS and a curing time of 90 days, UCS values of 2.84 and 3.59 MPa are obtained, respectively. These values are below the 4.64 MPa obtained result.

### 4.5. Relationship Between UCS at Short and Long Term

To establish the relationship between the short- and long-term UCS of this material, values obtained with the cylindrical samples at seven days were correlated with the results obtained for the prismatic sample tested at 90 days for the 10 manufactured batches.

The cylindrical samples obtained an average UCS value of 2.67 MPa with a standard deviation of 0.24 MPa. The prismatic samples obtained an average UCS value of 4.98 MPa with a standard deviation of 0.21 MPa 

The cylindrical samples showed approximately the same average UCS value that was employed for the mix design, whereas the prismatic samples obtained an average UCS value higher (7%) than the values obtained with the analysis of the UCS–FS relationship. This variability was due to the number of samples. 

**Figure 7:**
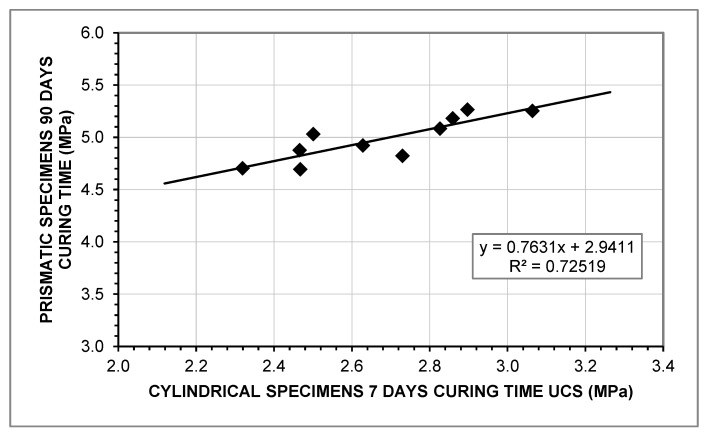
Relationship between UCS (7 days) and UCS (90 days).

The relationship between both UCS values can be obtained with a simple regression model, as shown in Figure 7.

The statistical analysis of the variables of the regression indicates that the linearity of the model is assured, since the *p*-value of the slope is below 0.05 and the intercept is significant with a 95% of confidence. The residuals follow a normal distribution, as the Kolmogorov–Smirnov test gives a value greater than 0.05 (0.8011). The plot of the residuals vs. predicted values confirm the homoscedasticity. The independence of the observations is assured by the Durbin–Watson statistic. Therefore, the regression model can be verified, correlating the relationship between the UCS at seven days (UCS_7_) and the UCS at long term (UCS_90_) through Equation (11).

(11) $${\mathrm{UCS}}_{90}=0.7631\times {\mathrm{UCS}}_{7}\;+\;2.9411$$

As in Equations (1)–(3), it is necessary to indicate the minimum threshold from which it is possible to state that the Equation (11) is valid. The same criteria established for Equations (1)–(3) are adopted for Equation (11), and, hence, the minimum UCS value at seven days must be 1.5 MPa.

For determining the evolution of the UCS with time, it is usual to employ the formula suggested by Lim and Zollinger [42] (Equation (8)), where UCS_t_ is the UCS value (in MPa) at any curing time t (in days) and UCS_28_ is the UCS at 28 days (MPa). However, Equation (8) uses the reference value of UCS at 28 days instead of UCS at seven days.

Linares [66] suggested a relationship between UCS_7_ and UCS_28_ as given by Equation (9). Hence, with an average value of 2.67 MPa for UCS_7_, by Equation (9), an average value of 3.89 MPa would be obtained for UCS_28_. Substituting this value in Equation (8), a value of 4.20 MPa will be obtained for UCS at 90 days. This value is below the obtained average value but is in the range of the average value ± standard deviation (4.64 ± 0.69 MPa).

## 5. Summary and Conclusions

Soil–cement manufactured in situ is a sustainable road construction technique that adds cement and water to the soil placed on or near the road to improve its characteristics, achieving a more resistant material. A higher proportion of cement would give greater resistances at long term but would increase the probability of cracking failure. Therefore, the amount of cement is usually limited to the one that guarantees the minimum value of the UCS at seven days.

This research was carried out for a soil with a maximum aggregate size of 40 mm, without organic materials and soluble sulfate. Added to it was 3.5% cement and 7% moisture content. With these values, a maximum dry density of 2.18 g/cm^3^ was obtained and UCS at seven days was 2.67 MPa.

It was demonstrated that both the UCS and density are variables that can be deployed to estimate the FS at long term. Introducing the density as a variable is a demand of the scientific community.

For establishing these correlations, 63 prismatic samples of 15 cm × 15 cm × 60 cm were manufactured and were tested in the four-point beam test for determining the FS. The two resulting prismatic parts were tested to determine their UCS, with a total of 125 samples.

From the results, the following conclusions can be obtained:

The models that best predict the Flexural Strength are:

- A linear multiple regression models, in which the dependent variable, FS at long term, is estimated from the UCS at long term and a dummy variable that depends on the compaction density (Model 3b).
- A model based on the Cobb–Douglas production function, where FS is the dependent variable and UCS at long term is the independent variable (Model 6b)
- A simple linear regression model where the FS is the dependent variable and UCS at long term is the independent variable (Model 1b)

Moreover, a relationship was determined between the UCS at seven days over cylindrical samples, employed to certify the quality of the soil–cement in situ, and the UCS at 90 days, by means of a simple linear regression. As this last model does not use the density as a variable, it is recommended to firstly use the Cobb–Douglas model and the simple linear regression for estimating the FS at long term from the UCS at seven days. However, for long-term analysis, where a more precise estimation is required, it is recommended to employ the multivariate linear regression model.

Obtained results validate the proposed models and, hence, the value of the FS at long term can be estimated from the values of the UCS at seven days, allowing applying correcting measures at early stages, if needed, such as recalculating the pavement structure with the real strength or rejecting it.

In all cases, the proposed models have an intercept, thus they can be used if the UCS value is greater than 1.5 MPa.

Although soil–cement has been widely used since the 1990s, the comparison of the proposed equations with other proposed ones confirms the necessity of following the advance of the knowledge in the characterization of soil–cement.

## Figures and Tables

**Table 1:** Cement properties.

Main Standardized Component	Value	Cement Standardized Specifications	Value
Clinker (K)	45–64%	Sulfate	≤3.5%
Silica fumes (D) ^1^	-	Initial setting time	≥75 min
Natural pozzolana (P) ^1^	-	Final setting time	≤720 min
Calcined natural pozzolans (Q) ^1^	-	Expansion	≤10 mm
Siliceous fly ash (V) ^1^	36–55%	UCS at 7 days	≥16 MPa
Calcareous fly ash (W) ^1^	-	UCS at 28 days	32.5 ≤ R ≤ 52.5 MPa
Minority components	0–5%	Puzzolanicity	8 to 15 days
Chlorides	≤0.10%	-	-

^1^ The sum of (D), (P), (Q), (V) and (W) for Cements CEM IV must be 36–55%.

**Table 2:** Requirements specified in various countries for the unconfined compressive strength (UCS) of soil–cement.

Country	UCS at 7 Days (MPa)
Spain [32]	2.5/2.1 ^1^
United Kingdom [30]	CBM1: 2.5–4.5
Australia [35]	≤3
New Zealand [31]	≤3
South Africa [34]	C2: 2–4
China [33]	3–5

^1^ For cements with a large amount of additions.

**Table 3:** Analysis of the simple linear regression with and without the intercept.

Analyzed item	Model 1a Without Intercept	Model 1b With Intercept
Slope	0.1854 (*p*-value < 0.0001)	0.1131 (*p*-value < 0.0001)
Intercept	-	0.3261 (*p*-value < 0.0001)
R^2^	0.3965	0.6953
Estimated standard error	0.1031	0.0735
Mean error	0.0809	0.0538
*p*-value (Kolmogorov–Smirnov)	0.4857	0.0822
Durbin–Watson	0.9916	1.3736 (*p*-value = 0.0002)

**Table 4:** Statistical analysis for the multiple regression models.

Analysed item	Model 3a Without Intercept	Model 3b With Intercept
Intercept	-	0.3230 (*p*-value < 0.0001)
UCS	0.1836 (*p*-value < 0.0001)	0.1124 (*p*-value < 0.0001)
Dummy	0.0417 (*p*-value = 0.0748)	0.0319 (*p*-value = 0.0564)
R^2^ fitted	0.6990	0.6995
Standard Error	0.1022	0.0728
F *p*-value (ANOVA)	<0.0001	<0.0001

**Table 5:** Statistical analysis for the Cobb–Douglas models.

Analysed item	Model 6b With Intercept	Model 8b With Intercept
Intercept	−1.0521 (*p*-value < 0.0001)	−1.0533 (*p*-value < 0.0001)
UCS	0.5808 (*p*-value < 0.0001)	0.5770 (*p*-value < 0.0001)
Dummy	-	0.0348 (*p*-value = 0.0974)
R^2^ fitted	0.7263	0.7302
Standard Error	0.0923	0.0916
F *p*-value (ANOVA)	<0.0001	<0.0001

**Table 6:** FS and UCS values compared with other authors’ formulae.

Author	Equation	Introduced Value	Estimated Value
Kersten [44]	FS = 0.2 × UCS (4)	UCS = 4.64 MPa	FS = 0.93 MPa
IECA-CEDEX [20]	FS = 0.2 × UCS (4)	UCS = 4.64 MPa	FS = 0.93 MPa
	FS = 0.25 × UCS (5)		FS = 1.16 MPa
Lim and Zollinger [42]	FS = 0.2 × UCS (4)	UCS = 4.64 MPa	FS = 0.93 MPa
	FS = 0.25 × UCS (5)		FS = 1.16 MPa
Ismail, et al [17]	USC = 1.475 × e^0.763·FS^ (6)	FS = 0.86 MPa, t = 90 days	USC = 2.84 MPa
	USC = 2.493 × FS^0.826^ × t^0.109^ (7)		USC = 3.59 MPa
Lim and Zollinger [42]	UCS_t_ = UCS_28_ × t/(2.5 + 0.9 × t) (8)	UCS_28_ = 3.89 MPa	UCS_90_ = 4.20 MPa
Linares [66]	UCS_28_ = 0.6947 × UCS_7_ + 2.0354 (9)	UCS_7_ = 2.67 MPa	UCS_28_ = 3.89 MPa

## References

[1] Nunes M.C.M., Bridges M.G., Dawson A.R. (1996). Assessment of secondary materials for pavement construction: Technical and environmental aspects. Waste Manag..

[2] López-Uceda A., Ayuso J., López M., Jimenez J., Agrela F., Sierra M. (2016). Properties of non-structural concrete made with mixed recycled aggregates and low cement content. Materials.

[3] López-Uceda A., Ayuso J., Jiménez J., Agrela F., Barbudo A., De Brito J. (2016). Upscaling the use of mixed recycled aggregates in non-structural low cement concrete. Materials.

[4] Pérez-Acebo H., Linares-Unamunzaga A., Abejón R., Rojí E. (2018). Research trends in pavement management during the first years of the 21st century: A bibliometric analysis during the 2000–2013 period. Appl. Sci..

[5] Salas M.Á., Pérez-Acebo H., Calderón V., Gonzalo-Orden H. (2018). Bitumen modified with recycled polyurethane foam for employment in hot mix asphalt. Ingeniería e Investigación.

[6] Díaz J., Linares A., González D., Gonzalo-Orden H. In situ pavement recycling with cement design. Proceedings of the 4th European Pavement and Asset Management Conference (EPAM).

[7] Amato G., Campione G., Cavaleri L., Minafò G., Miraglia N. (2012). The use of pumice lightweight concrete for masonry applications. Mater. Struct..

[8] Pérez-Acebo H., Bejan S., Gonzalo-Orden H. (2017). Transition probability matrices for flexible pavement deterioration models with half-year cycle time. Int. J. Civ. Eng..

[9] Bejan S., Pérez-Acebo H. (2016). Modeling the dynamic interaction between a vibratory-compactor and ground. Rom. J. Acoust. Vib..

[10] Islam S., Haque A., Bui H. (2016). 1-D compression behaviour of acid sulphate soils treated with alkali-activated slag. Materials.

[11] Zhang Y., Guo Q., Li L., Jiang P., Jiao Y., Cheng Y. (2016). Reuse of boron waste as an additive in road base material. Materials.

[12] Jing P., Nowamooz H., Chazallon C. (2017). Effect of anisotropy on the resilient behaviour of a granular material in low traffic pavement. Materials.

[13] Molenaar A.A.A. (2007). Lecture Notes CT 4860 Structural Pavement Design. Design of Flexible Pavements.

[14] Adaska W.S. (1997). State-of-the-art report on soil-cement. Mater. J..

[15] Wu P., Molenaar A.A.A., Houben I.L. (2011). Cement-Bound Road Base Materials.

[16] Xuan D.X., Houben L.J.M., Molenaar A.A.A., Shui Z.H. (2012). Mechanical properties of cement-treated aggregate material—A review. Mater. Des..

[17] Ismail A., Baghini M.S., Karim M.R., Shokri F., Al-Mansob R.A., Firoozi A.A., Firoozi A.A. (2014). Laboratory investigation on the strength characteristics of cement-treated base. AMM Appl. Mech. Mater..

[18] Otte E. (1978). A Structural Design Procedure for Cement-Treated Layers in Pavements.

[19] Bell F.G. (1993). Engineering Treatment of Soils.

[20] IECA-CEDEX (2003). Manual de Firmes con Capas Tratadas con Cemento.

[21] PCA (1992). Soil-Cement Laboratory Handbook.

[22] Xuan D.X. (2009). Literature Review of Research Project: Structural Properties of Cement Treated Materials.

[23] Arulrajah A., Ali M.M.Y., Piratheepan J., Bo M.W.M.A. (2012). Geotechnical properties of waste excavation rock in pavement subbase applications. J. Mater. Civ. Eng..

[24] Disfani M.M., Arulrajah A., Haghighi H., Mohammadinia A., Horpibulsuk S. (2014). Flexural beam fatigue strength evaluation of crushed brick as a supplementary material in cement stabilized recycled concrete aggregates. Constr. Build. Mater..

[25] Garach L., López M., Agrela F., Ordóñez J., Alegre J., Moya J. (2015). Improvement of bearing capacity in recycled aggregates suitable for use as unbound road sub-base. Materials.

[26] Jofré C., Díaz J. Soilcement subbases: Mix in place vs. mix in plant. Proceedings of the 2nd International symposium on subgrade stabilisation and in situ pavement recycling using cement, TREMTI.

[27] Baghini M.S., Ismail A., Firoozi A.A. (2016). Physical and mechanical properties of carboxylated styrene-butadiene emulsion modified portland cement used in road base construction. J. Appl. Sci..

[28] Lv S., Liu C., Lan J., Zhang H., Zheng J., You Z. (2018). Fatigue equation of cement-treated aggregate base materials under a true stress ratio. Appl. Sci..

[29] Hong S. (2017). Influence of curing conditions on the strength properties of polysulfide polymer concrete. Appl. Sci..

[30] British Standards Institution (BSI) (1990). BS-1924-1: Stabilized materials for civil engineering purposes. General Requirements, Sampling, Sample Preparation and Tests on Materials before Stabilization.

[31] CCANZ (2008). IB-89. Cement Stabilisation.

[32] Ministerio de Fomento (2015). Pliego de Prescripciones Técnicas Generales Para Obras de Carretera y Puentes (PG-3).

[33] Ministry of Communications (MOC) (2000). JTJ034-2000: Technical Specifications for Construction of Highway Road Bases.

[34] Molenaar A.A.A. (1998). Road Materials-Part II: Soil Stabilization (Lecture CT4850).

[35] TMR (2010). Technical Standard MRTS08 Plant-Mixed Stabilised Pavements Using Cement or Cementitious Blends: Transport and Main Roads Specifications.

[36] Kolias S., Williams R.I.T. (1978). Cement-Bound Road Materials: Strength and Elastic Properties Measured in the Laboratory.

[37] Han Y.J., Oh S.K., Kim B. (2017). Effect of load transfer section to toughness for steel fiber-reinforced concrete. Appl. Sci..

[38] Mansoor J., Shah S., Khan M., Sadiq A., Anwar M., Siddiq M., Ahmad H. (2018). Analysis of mechanical properties of self compacted concrete by partial replacement of cement with industrial wastes under elevated temperature. Appl. Sci..

[39] Yeo R. (2008). Construction Report for Cemented Test Pavements—Influence of Vertical Loading on the Performance of Unbound and Cemented Materials.

[40] Reeder G.D., Harrington D., Ayers M.E., Adaska W.S. (2017). Guide to Full-Depth Reclamation (FDR) with Cement.

[41] ASTM (2017). D1632-17: Standard Practice for Making and Curing Soil-Cement Compression and Flexure Test Specimens in the Laboratory.

[42] Lim S., Zollinger D.G. (2003). Estimation of the compressive strength and modulus of elasticity of cement-treated aggregate base materials. Transp. Res. Rec..

[43] Thompson M.R. (1986). Mechanistic Design Concepts for Stabilized Base Pavements.

[44] Kersten M.S. (1961). Soil Stabilization with Portland Cement.

[45] Brown S.F. (1979). Design of pavements with lean-concrete bases. Transp. Res. Rec..

[46] Williams R.I.T. (1986). Cement-Treated Pavements: Materials, Design and Construction.

[47] ASTM (2017). D4318-17: Standard Test Methods for Liquid Limit, Plastic Limit, and Plasticity Index of Soils.

[48] ASTM (2014). D2974-14: Standard Test Methods for Moisture, Ash, and Organic Matter of Peat and Other Organic Soils.

[49] ASTM (2014). C1580-15: Standard Test Method for Water-Soluble Sulfate in Soil.

[50] ASTM (2014). D2419-14: Standard Test Method for Sand Equivalent Value of Soils and Fine Aggregate.

[51] ASTM (2011). D2487-11: Standard Practice for Classification of Soils for Engineering Purposes (Unified Soil Classification System).

[52] AENOR (2011). UNE-EN 197-1. Parte 1: Composición, Especificaciones y Criterios de Conformidad de los Cementos Communes.

[53] AENOR (1994). UNE 103-501-94: Geotecnia: Ensayo de Compactación Proctor Modificado.

[54] ASTM (2012). D1557-12e1: Standard Test Methods for Laboratory Compaction Characteristics of Soil Using Modified Effort (56,000 ftlbf/ft3 (2700 kN-m/m^3^)).

[55] Linares-Unamunzaga A., Gonzalo-Orden H., Minguela J., Pérez-Acebo H. (2018). New procedure for compacting prismatic specimens of cement-treated base materials. Appl. Sci..

[56] AENOR (2009). UNE-EN 12390-3. Parte 3: Determinación de la Resistencia a Compresión de Probetas.

[57] Lacidogna G., Piana G., Carpinteri A. (2017). Acoustic Emission and Modal Frequency Variation in Concrete Specimens under Four-Point Bending. Appl. Sci..

[58] Díaz J. (2011). El Estudio de Comportamiento de los Firmes Reciclados In Situ con Cemento. Ph.D. Thesis.

[59] AENOR (2009). UNE-EN 12390-5. Parte 5: Resistencia a Flexión de Probetas.

[60] AENOR (2003). Norma UNE-EN 13286-41. Mezclas de Áridos sin Ligante y con Conglomerante Hidráulico. Parte 41: Método de Ensayo Para la Determinación de la Resistencia a la Compresión de las Mezclas de Áridos con Conglomerante Hidráulico.

[61] CEDEX (1990). Norma NLT-305/90. Resistencia a Compresión Simple de Materiales Tratados con Conglomerantes Hidráulicos.

[62] Wonnacott T.H., Wonnacott R. (1977). Introductory Statistics.

[63] ASTM (2012). D1635/D1635M-12: Standard Test Method for Flexural Strength of Soil-Cement Using Simple Beam with Third-Point Loading.

[64] Montgomery D.C., Peck E.A., Vining G.G. (2012). Introduction to Linear Regression Analysis.

[65] Darlingon R.B., Hayes A.F. (2017). Regression Analysis and Linear Models. Concepts, Applications and Implementation.

[66] Linares A. (2015). Metodología Para el Avance en la Caracterización del Suelocemento de Aplicación en Firmes Semirrígidos. Ph.D. Thesis.

